# The histone deacetylase inhibitor Scriptaid targets G-quadruplexes

**DOI:** 10.1098/rsob.240183

**Published:** 2025-02-19

**Authors:** Victoria Sanchez-Martin, Dusan Ruzic, Maria J. Tello-Lopez, Andrea Ortiz-Morales, Javier Murciano-Calles, Miguel Soriano, Katarina Nikolic, Jose Antonio Garcia-Salcedo

**Affiliations:** ^1^ GENYO, Centre for Genomics and Oncological Research, Pfizer/University of Granada/Andalusian Regional Government, Granada 18016, Spain; ^2^ Microbiology Unit, University Hospital Virgen de las Nieves, Granada 18014, Spain; ^3^ Instituto de Investigación Biosanitaria de Granada (ibs.GRANADA), Granada 18012, Spain; ^4^ Department of Biochemistry, Molecular Biology III and Immunology, University of Granada, Granada 18016, Spain; ^5^ Department of Pharmaceutical Chemistry, Faculty of Pharmacy, University of Belgrade, Belgrade 11221, Serbia; ^6^ Center for Intensive Mediterranean Agrosystems and Agri-Food Biotechnology (CIAIMBITAL), University of Almeria, Almeria 04001, Spain; ^7^ Department of Physical Chemistry, Unit of Excellence for Chemistry Applied to Biomedicine and the Environment, and Institute of Biotechnology, University of Granada, Granada 18071, Spain

**Keywords:** G-quadruplex, histone deacetylase inhibitor, transcription, RNA polymerase I, colorectal cancer

## Abstract

Scriptaid is a chemical compound with anti-tumoural effects due to its role as a histone deacetylase inhibitor. Despite sharing part of the chemical structure with other ligands of G-quadruplexes (G4s), the interaction of Scriptaid with G4s has not been explored before. We synthesized Scriptaid and screened its cytotoxic activity in cellular models of colorectal cancer (CRC). We extensively evaluated its biological activity by cell cycle, immunofluorescence, qRT-PCR and Western blot experiments. To identify the G4 targets of Scriptaid, we conducted a panel of binding assays. Here, we show that Scriptaid induced cytotoxicity, cell cycle arrest and nucleolar stress in CRC cells. Moreover, Scriptaid impaired RNA polymerase I (Pol I) transcription, stabilized G4s and caused DNA damage. Finally, we disclose that these effects were attributable to the binding of Scriptaid to G4s in ribosomal DNA. In conclusion, our work reveals that a primary impact of Scriptaid on human cells is the interaction with G4s.

**Figure:**
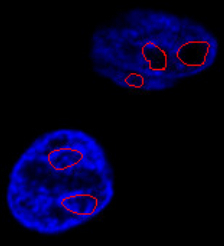

## 1. Background

Tumour initiation and progression are driven by genetic alterations combined with epigenetic abnormalities. In this regard, epigenetic modulation is exploited for cancer treatment [1]. Conventional therapy in combination with epigenetic therapy exhibits significant beneficial effects in solid tumours [2]. In particular, histone deacetylases (HDACs) are primarily responsible for the removal of acetyl groups from amino terminal lysine residues of histones, displaying a key role in the epigenetic regulation of gene expression [3]. In addition, numerous non-histone proteins are modified by acetylation rendering pleiotropic effects [4]. In this sense, HDAC inhibition has emerged as a potential therapeutic strategy to reverse the epigenetic alterations associated with cancer [5].

HDAC inhibitors (HDACIs) have moved rapidly in clinical applications. Vorinostat (SAHA, Zolinza) was approved for the treatment of T-cell lymphoma by FDA in 2006 [6]. From then on, a range of HDACIs has been explored for cancer therapy [7]. In colorectal cancer (CRC), a novel HDAC1/2 inhibitor has been considered as a promising lead drug [8]. The chemical structure of HDACIs consists of three parts: a zinc chelating group; a spacer group, which is generally hydrophobic; and a capping (CAP) group which is generally aromatic and confers specificity [9]. Interestingly, the synthetic analogue Scriptaid was identified as an HDACI by high-throughput transcriptional screening of a library of compounds [10]. Scriptaid is a pan-HDACI that efficiently inhibits nuclear HDAC isoforms (HDAC1 IC_50_ = 0.2 μM; HDAC2 IC_50_ = 0.7 μM; HDAC3 IC_50_ = 0.1 μM and HDAC8 IC_50_ = 0.3 μM) as well as cytoplasmic class IIb HDACs (HDAC6 IC_50_ = 0.01 μM and HDAC10 IC_50_ = 0.3 μM) [11]. Multiple studies have reported the anti-tumoural activity of Scriptaid [12]. However, most of them are focused on its growth inhibitory as well as apoptosis-inducing activity. Identifying the key molecular actions that dictate the biological response to Scriptaid remains a challenge.

Scriptaid is a 1,8-naphthalimide derivative sharing part of the chemical structure with naphthalene diimides, which are known to display DNA-binding properties [13]. Particularly, in a previous work, we disclosed that naphthalene diimides bind to G-quadruplexes (G4s) on ribosomal DNA producing an anti-tumoural response in CRC [14]. DNA G4s are four-stranded, non-canonical secondary structures folded in guanine-rich sequences [15], which have been demonstrated to be promising targets for anti-tumoural therapy [16,17]. Nevertheless, the interaction of Scriptaid with DNA G4s has been previously explored in just one study [18], where authors reported that Scriptaid does not bind to a telomeric G4, but they did not investigate the interaction with additional G4 sequences. In this context, we aimed to analyse the anti-tumoural effects of Scriptaid and decipher its underlying biological mechanism using the CRC cellular model which is available in our laboratory.

## 2. Material and methods

### 2.1. Synthesis of Scriptaid

Scriptaid was synthesized in accordance with literature procedure [19]. 6-(1),3-Dioxo-1H,3H-benzo[de]isoquinolin-2-yl)-hexanoic acid (2) a mixture of 1.8-naphthalic anhydride (1.0 g, 1.0 equiv, 0.0550 mol) and 6-aminohexanoic acid (0.728 g, 1.1 equiv, 0.7282 mol) in dimethylformamide (20 ml) was refluxed for 6 h. The mixture was poured into ice water to precipitate out a solid. Crystals were collected by filtration, washed with water and dried to afford compound 2 (1.16 g, 73.9%) as a white solid. 6-(1),3-Dioxo-1H,3H-benzo[de]isoquinolin-2-yl)-hexanoic acid hydroxyamide (Scriptaid) compound 2 (100 mg, 1 equiv, 0.3212 mmol) was disolved in CH_2_Cl_2_ (3 ml) and EDCI•HCl (86.2 mg, 1.4 equiv, 0.4496 mmol), anhydrous HOBt (78.11 mg, 1.8 equiv, 0.5781 mmol), Et_3_N (58.50 mg, 1.8 equiv, 0.5781 mmol) and O-(Tetrahydro-2H-pyran-2-yl)hydroxylamine (56.44 mg, 1.5 equiv, 0.4818 mmol) were added. The mixture was stirred at 40°C overnight, transferred to separatory funnel, washed with brine (3 × 5 ml), dried over Na_2_SO_4_ and concentrated in vacuo. Crude product was dissolved in 2-propanol (3 ml), p-toluenesulfonic acid (18.94 mg, 0.3 equiv, 0.0995 mmol) was added. The reaction mixture was stirred overnight at room temperature (RT). Solvent was evaporated in vacuo and Scriptaid was purified by flash chromatography (PE/EtOAC = 2/1 and DCM/MeOH 95/5, v/v) to afford Scriptaid (40.5 mg, 38.6%).

### 2.2. Cell culture

A375, CRL1790, SW480, HT29 and HCT116 cell lines were acquired from American Type Culture Collection (ATCC). Cells were grown at 37°C, 5% CO_2_ in medium supplemented with 10% fetal bovine serum (10270106, Gibco), 0.03% L-Glutamine (G8540, Sigma Aldrich), 10 mg/ml penicillin (P0781, Sigma Aldrich), 10 mg/ml streptomycin (P0781, Sigma Aldrich) and 100 mg/ml amphotericin (A2942, Sigma Aldrich), following the ATCC guidelines. All procedures were performed under aseptic conditions, and biosafety requirements were met. Experiments were carried out with cells at 12−15th passage number.

### 2.3. Cytotoxic assay

Resazurin Fluorimetric Assay (R7017, Sigma Aldrich) was used to screen cytotoxic activity in triplicate. Cells previously seeded into 96-well plates (8·10^3^ cells per well) were treated for 48 h with increasing concentrations of Scriptaid that ranged from 5·10^−1^ µM to 5·10^2^ µM. A negative control with dimethyl sulfoxide (DMSO) was included. Fluorescence was measured using Nanoquant Infinite M200 Pro multi-plate reader (Tecan). IC_50_ values were determined by nonlinear regression with Graphpad (Prism).

### 2.4. Cell cycle analysis

Flow cytometry with propidium iodide (PI) (P4864, Sigma Aldrich) was employed to analyse cell cycle distribution. SW480 cells (10^6^) were previously seeded into 10 cm culture dishes and treated with Scriptaid at IC_50_ concentration for 24 h or the vehicle (DMSO) as control. Cells were then ice-cold 70% ethanol-fixed on ice and stained with 0.04 mg ml^−1^ PI and 0.1 mg ml^−1^ ribonuclease A (19101, Qiagen). Cell cycle analysis was achieved by an analytical DNA flow cytometer (FACSVerse, BD Biosciences) and FlowJo software.

### 2.5. Immunofluorescence experiments

Cells were seeded into 13 mm circular coverslips (80,000 cells/coverslip) that were placed in 24-well plates and subsequently treated with different experimental conditions. Fixation was performed with 4% (v/v) paraformaldehyde (P6148, Sigma Aldrich) for 10 min at RT, permeabilization with 0.1% (v/v) Triton-X100 (T8787, Sigma Aldrich) for 10 min and blocking with 10% bovine serum albumin (A7906, Sigma Aldrich), 0.5% (v/v) Triton-X100 for 30 min at RT. Then, primary antibodies were incubated for 1 h at RT and secondary antibodies for 30 min at 4°C with darkness. All coverslips were mounted onto poly-L-lysine slides (J2800AMNZ, Thermo Scientific) with Vectashield (H-1200, Vector) that included DAPI (4’,6-diamidino−2-phenylindole) for nuclear counterstain. Images were acquired with a Confocal Zeiss LSM 710 inverted microscope using a 63 × immersion objective, but zoom-in images are displayed to ease the visualization of the reported changes. The images were captured from randomly selected fields of view. Antibodies used are listed in electronic supplementary material, table S1. Particularly, the BG4 antibody was expressed and used for immunofluorescence according to the protocol previously described [20]. Nuclear fluorescence quantification was performed by Fiji analysis.

### 2.6. qRT-PCR

Trizol Reagent (15596, Invitrogen) was used to isolate total RNA. Reverse transcription was conducted with RevertAid First Strand cDNA Synthesis Kit (K1622, Thermo Fisher Scientific) using random primers following the manufacturer’s protocol. Quantitative PCR (qPCR) was performed with SYBR Green (4309155, Thermo Fisher Scientific) on 7900HT Fast Real-time PCR System (Applied Biosystems). Cycling conditions were: 95°C for 10 min, 40 cycles of 15 s at 95°C and 1 min at 60°C and a final dissociation stage. Target mRNA levels were normalized (ΔCt) to actin as housekeeping control, and fold change was determined by the 2^-ΔΔCT^ method. Experiments were performed in biological triplicate. CX5461 (HY-13323, MedChemExpress) is a well-known Pol I inhibitor that was used as a positive control for Pol I inhibition. The sequence of primers used is listed in electronic supplementary material, table S2.

### 2.7. Western blot

RIPA lysis buffer containing 1% PMSF (P7626, Sigma Aldrich) and 1% protease inhibitor cocktail (P8340, Sigma Aldrich) was used to obtain the protein extract. Protein content was loaded on 12% SDS-polyacrylamide gels (1610148, Bio-Rad), separated by electrophoresis and wet-transferred to nitrocellulose membranes (66485, Pall corporation). Membranes were blocked with 5% semi-skimmed milk, incubated overnight at 4°C with primary antibodies, and then with Horseradish Peroxidase (HRP)-labelled secondary antibodies for 1 h at RT. Upon incubation with luminol solution (1705060, Bio-Rad), chemiluminiscence was measured using Image Quant LAS 4000 (GE Healthcare Life Sciences). Protein levels were quantified by ImageJ software. Experiments were performed in biological triplicate. Antibodies used are included in electronic supplementary material, table S1.

### 2.8. Thioflavin T competition assay

Cells were seeded into 13 mm circular coverslips that were placed in 24-well plates (8·10^4^ cells per well) and treated with vehicle or Scriptaid 1 µM for 3 h. Then, cells were cold methanol-fixed for 10 min, rinsed with PBS twice and incubated with 5 µM thioflavin T (ThT) (T3516, Sigma-Aldrich) for 15 min. PI was added for nuclear counterstain. Finally, cellular nuclei were visualized using confocal microscopy as previously described [21].

### 2.9. BG4 chromatin immunoprecipitation

Chromatin immunoprecipitation (ChIP) with BG4 antibody was performed as previously described [22]. Chromatin was extracted, sheared to 100−500 bp using Bioruptor Plus (Diagenode) and treated with 20 μg ml^−1^ RNase A (19101, Qiagen) to remove RNA G4s and DNA–RNA G4 hybrids. Pierce Protein A/G Magnetic Beads (88802, Thermo Fisher Scientific) were used to capture anti-FLAG antibody (F1804, Sigma Aldrich) following manufacturer’s instructions. Per ChIP reaction, a total amount of 500 ng of DNA was immunoprecipitated with 500 ng of BG4 and subsequently collected with the magnetic beads. Finally, DNA was purified using phenol:chloroform:isoamyl alcohol (25 : 24 : 1) protocol (P2069, Sigma). Experiments were conducted in biological triplicate.

### 2.10. G4s prefolding

G4 oligonucleotides harboured in *5’ETS* of ribosomal DNA [14] were acquired from Sigma Aldrich. All G4 oligonucleotides were dissolved in G4s buffer [10] mM potassium phosphate buffer containing 100 mM potassium chloride at pH 7.0), heated at 95°C for 10 min, slowly tempered to RT and stored at 4°C. The sequence of G4 oligonucleotides used is listed in electronic supplementary material, table S2.

### 2.11. Fluorescent intercalator displacement assay

The fluorescent intercalator TOPRO3 (T3605, Thermo Scientific) at 5 μM was incubated with 10 μM prefolded G4s and Scriptaid 10 μM in 96-well plates. TOPRO3 emission profile was monitored from 650 to 800 nm with a multi-plate reader upon excitation at 642 nm. All assays were conducted in triplicate. Fluorescence was calculated as follows: % fluorescence = *A*/*B*·100; where *A* is the fluorescence value in presence of Scriptaid and *B* corresponds to the fluorescence value in Scriptaid-free controls.

### 2.12. PCR-stop assay

PCR-stop assay was performed as described before [14] using a test oligonucleotide with the G4 sequence and a partially complementary oligonucleotide. Sequence of primers used is listed in electronic supplementary material, table S2. Increasing amounts of Scriptaid from 0 to 100 μM were used and vehicle DMSO as control. Amplification was conducted in a Veriti Thermal Cycler (Applied Biosystems) with the following cycling conditions: 95°C for 15 min, 30 cycles of 95°C for 30 s, 58°C for 30 s and 72°C for 30 s. Finally, PCR products were resolved on 3% agarose gel in 1X TBE (100 mM Tris base, 100 mM boric acid, 2 mM EDTA) and stained with GelGreen (41005, Biotium). Gel images were acquired on ImageQuant LAS 4000. Per concentration, three independent reactions were conducted, and representative lanes were displayed.

### 2.13. Circular dichroism

Circular dichroism (CD) experiments were performed as previously described [23]. CD spectra were recorded at 25°C on a JASCO 715 CD spectropolarimeter in presence of G4s buffer. The cuvette path length was 0.1 cm. Prefolded G4 DNA at 10 μM was incubated overnight with and without Scriptaid at 100 μM to register the spectra. The wavelength range was 230−700 nm with 100 nm/min as scan speed. For each measurement, three accumulation spectra were averaged. For melting studies, prefolded G4 DNA at 10 μM was incubated with and without Scriptaid at 100 μM. Thermal melting was monitored at 265 nm from 25°C to 98°C at a heating rate of 1°C/min. The melting temperatures were determined from the sigmoidal curve fit using the thermodynamic equations referred to a model considering two states (native and unfolded), the so-called two-state model. The sequence of G4 oligonucleotide used is listed in electronic supplementary material, table S2.

### 2.14. Ultraviolet−visible titration

The spectra of ultraviolet−visible (UV−vis) absorption were acquired with a 0.3 cm cuvette in a Varian Cary 50 UV−vis spectrophotometer at 25°C. The wavelength range used was 240−330 nm. In presence of G4s buffer, 5 μM of the prefolded G4 DNA was prepared, and a concentrated solution of Scriptaid [1] mM) was routinely added with a Hamilton syringe and subsequently mixed with a pipette. The UV−vis spectrum was recorded after each addition of Scriptaid. In total, the final ratio of G4 DNA:Scriptaid was 1:50. For the blank, the same experiment with the successive additions was repeated beginning just with buffer in the cuvette. Then, each titration spectrum was subtracted from its corresponding blank. Experiments were performed in triplicate. Sequence of G4 oligonucleotide used is listed in electronic supplementary material, table S2. The dissociation constant (Kd) was determined at 260 nm using the following formula:


$$A=A_{m}+(A_{ml}-A_{m})\times \frac{(M+L+K_{d})-\sqrt{(M+L+K_{d}{)}^{2}}-4\times M\times L}{2\times M},$$

where A is the absorbance, A_m_ is the absorbance of DNA, A_ml_ is the absorbance of DNA in presence of the ligand, M is the total concentration of DNA, L is the total concentration of ligand and K_d_ is the dissociation constant.

### 2.15. Statistical analysis

Statistical significance was analysed using Student’s two-tailed *t*‐test. For all tests, *p*-values below 0.05 were considered significant and represented as follows: *p < 0.05; **p < 0.01; and ***p < 0.001.

## 3. Results

### 3.1. Scriptaid is synthesized and shows cytotoxic activity in CRC

**Figure 1.**
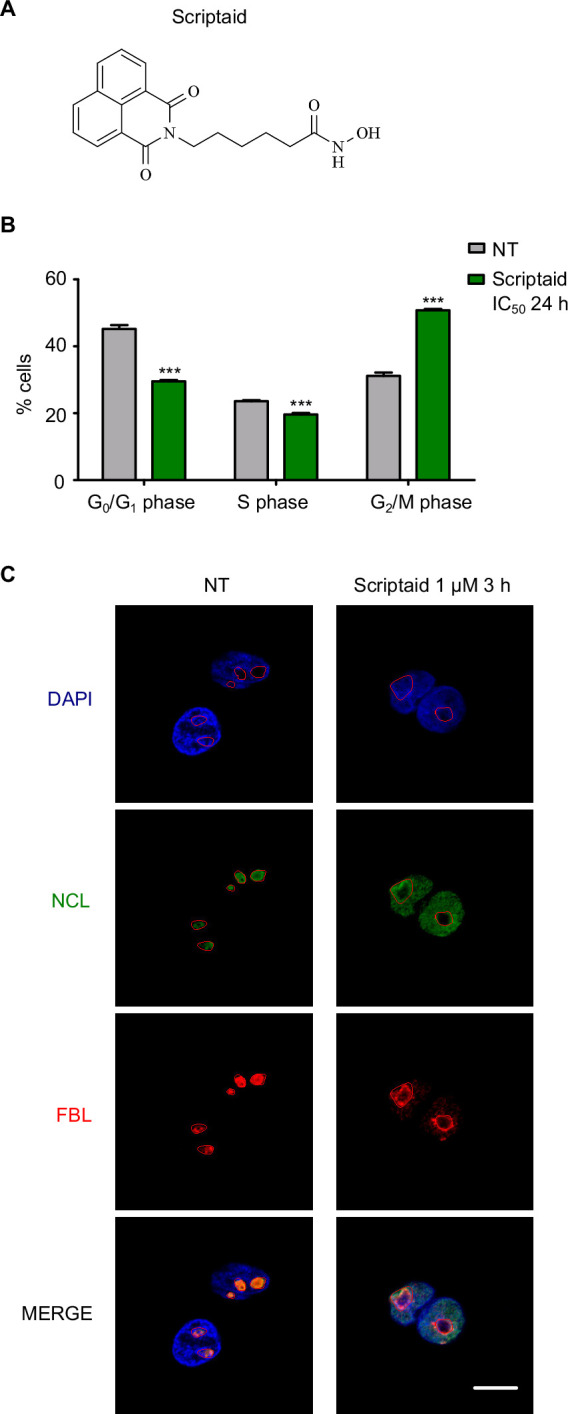
Scriptaid induces cell cycle arrest and nucleolar stress. (A) Chemical structure of Scriptaid. (B) Propidium iodide flow cytometry analysis of SW480 cells treated with DMSO (non-treated, NT) or Scriptaid at IC_50_ for 24 h. Stacked bar graph illustrating cell cycle distribution is displayed. (C) SW480 cells were treated with DMSO (NT) or Scriptaid 1 µM for 3 h and immuno-stained to detect nucleolin (NCL) and fibrillarin (FBL) by immunofluorescence. DAPI was used for nuclear counterstain and the red lines outline the nucleoli. Merged images are shown below. Scale bar, 10 µm.

Scriptaid (figure 1A) was synthesized and characterized (electronic supplementary material, figure S1) according to literature procedure [19]. In order to evaluate its therapeutic potential in CRC, we determined the half-maximal inhibitory concentration (IC_50_) both in CRL1790 colon epithelial, non-tumoural cells, and in SW480 colorectal adenocarcinoma cells. Cells were treated with increasing concentrations of Scriptaid ranging from 5·10^-1^ to 5·10^2^ µM for 48 h. As result, Scriptaid induced cell cytotoxicity in both cell lines (table 1; electronic supplementary material, figure S2). Then, the selectivity index was determined as the ratio of IC_50_ for non-tumoural and tumoural cells. Interestingly, selectivity indexes were higher than 2.0, suggesting that tumoural cells were over twice more sensitive than non-tumoural ones to treatment with Scriptaid. Considering that Scriptaid met the selection criteria for anticancer agents (IC_50_ lower than 30 µM and selectivity index greater than 2) that had been previously established [24], we confirmed its anti-tumoural potential. Selective cytotoxic activity of Scriptaid was also demonstrated in other CRC cell lines including HCT116 and HT29, regardless of *TP53* mutational status (table 1; electronic supplementary material, figure S2). Based on these results, Scriptaid was considered as a leading anti-tumoural candidate in CRC for subsequent studies with SW480 cells.

**Table 1.** IC_50_ values and selectivity index for Scriptaid in different cell lines. IC_50_ values show Scriptaid concentration inhibiting cell growth by 50% and are represented as mean ± s.d.. Selectivity index is the ratio of IC_50_ values between non-tumoural (CRL1790) and the respective CRC cell lines. Experiments were performed in biological triplicate.

cell line	IC50 (µM)	selectivity index
**CRL1790** (non-tumoural)	128.8 ± 5.4	—
**SW480** (CRC, TP53 mut)	9.6 ± 1.3	13.5
**HT29** (CRC, TP53 mut)	8.9 ± 0.6	13.5
**HCT116** (CRC, TP53 wt)	42.8 ± 2.1	3.0

### 3.2. Scriptaid induces cell cycle arrest and nucleolar stress

The effect of Scriptaid on cell cycle was analysed by flow cytometry with PI. Scriptaid at IC_50_ induced a significant arrest at G2/M phase on SW480 cells treated for 24 h (figure 1B). Next, we explored the nucleolar status considering the similarities on chemical structure between Scriptaid and naphthalene diimides, which were reported to cause nucleolar disassembly in our previous work [14]. To decipher the upstream effects of Scriptaid, we reduced the concentration to 1 μM and the time of exposure to 3 h for further studies, in the same manner as our previous work with naphthalene diimides [14]. At these experimental conditions, Scriptaid still inhibited HDACs since histone H3 was significantly acetylated (K27), but the acetylation signal was accumulated at nuclear periphery (electronic supplementary material, figure S3). Then, we performed an immunofluorescence of SW480 cells treated with vehicle or Scriptaid 1 µM for 3 h to determine the intracellular localization of nucleolar proteins [25]. To define the nucleoli in every cell, we focused on DAPI staining considering that the nucleolus appears as a black cavity in the nucleus because of a lower concentration of DNA in the nucleolus compared with the surrounding nucleoplasm [26]. Additionally, nucleolin (NCL) staining guided to outline the nucleoli since NCL is the most abundant phosphoprotein of the nucleolus [27]. In particular, Scriptaid caused a remarkable nucleolar stress characterized by the translocation of nucleolin from nucleolus to nucleoplasm, as well as the segregation of fibrillarin (FBL) to nucleolar periphery caps, although their protein levels were not affected (figure 1C; electronic supplementary material, figure S4). These effects were indicative of nucleolar stress.

### 3.3. Scriptaid impairs Pol I transcription of ribosomal DNA

**Figure 2.**
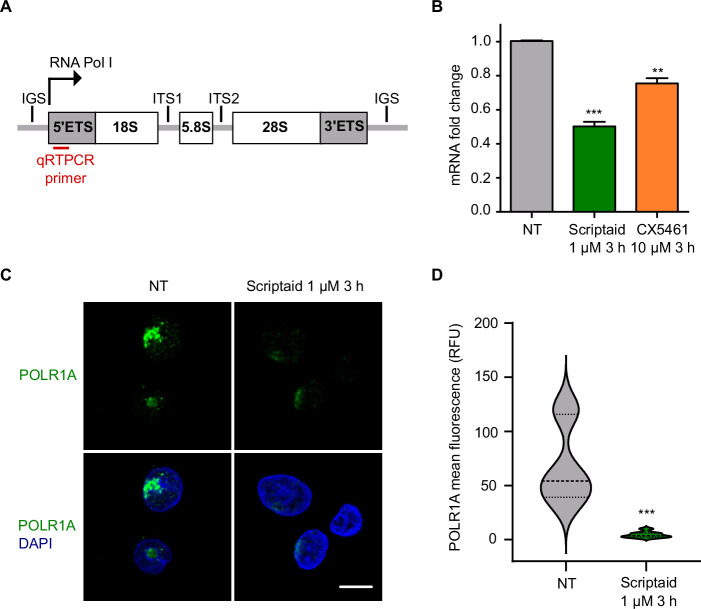
Scriptaid impairs Pol I transcription. (A) Schematic representation from the arrangement of human ribosomal DNA with repeated units, each one containing an RNA coding region (box) and an intergenic spacer (line). Location of qRT-PCR primers used for this study is represented in red. (B) SW480 cells were treated with vehicle DMSO (NT), Scriptaid 1 µM or CX5461 10 µM for 3 h and ribosomal DNA transcription was analysed by qRT-PCR with primers for short-lived *5’ETS*. Experiments were performed in biological triplicate. (C) SW480 cells were treated with DMSO (NT) or Scriptaid 1 µM for 3 h and immuno-stained to detect Pol I catalytic subunit A (POLR1A) by immunofluorescence. Merged image with DAPI for nuclear counterstain is shown below. Scale bar, 10 µm. (D) Nuclear POLR1A fluorescence quantification from cells in (C) by Fiji analysis (*n* > 50).

Disruption of the nucleolar structure usually occurs when rRNA transcription is blocked [25]. Therefore, we aimed to determine whether Scriptaid affected RNA polymerase I (Pol I)-transcriptional output. We measured the short-lived 5’ external transcribed spacer (*5’ETS*) of the pre-rRNA by qRT-PCR, considering that *5’ETS* abundance reflects the rRNA synthesis rate [28] (figure 2A). We observed a significant decrease in the levels of *5’ETS* transcripts in SW480 cells treated with Scriptaid 1 µM for 3 h, as occurred with CX5461 that was used as control for Pol I inhibition (figure 2B). In some cases, impairment of Pol I transcription upon treatment with Pol I inhibitors may lead to Pol I degradation [14]. In fact, an important loss of Pol I catalytic subunit A (POLR1A) in SW480 cells treated with Scriptaid 1 µM for 3 h was observed by immunofluorescence experiments (figure 2C). POLR1A mean fluorescence quantification confirmed a significant decrease in POLR1A after Scriptaid treatment (figure 2D). In addition, Western blot also demonstrated this significant degradation of POLR1A after incubation with Scriptaid (electronic supplementary material, figure S5).

### 3.4. Scriptaid stabilizes G4s inducing DNA damage and affects Pol II transcription of several oncogenes

**Figure 3.**
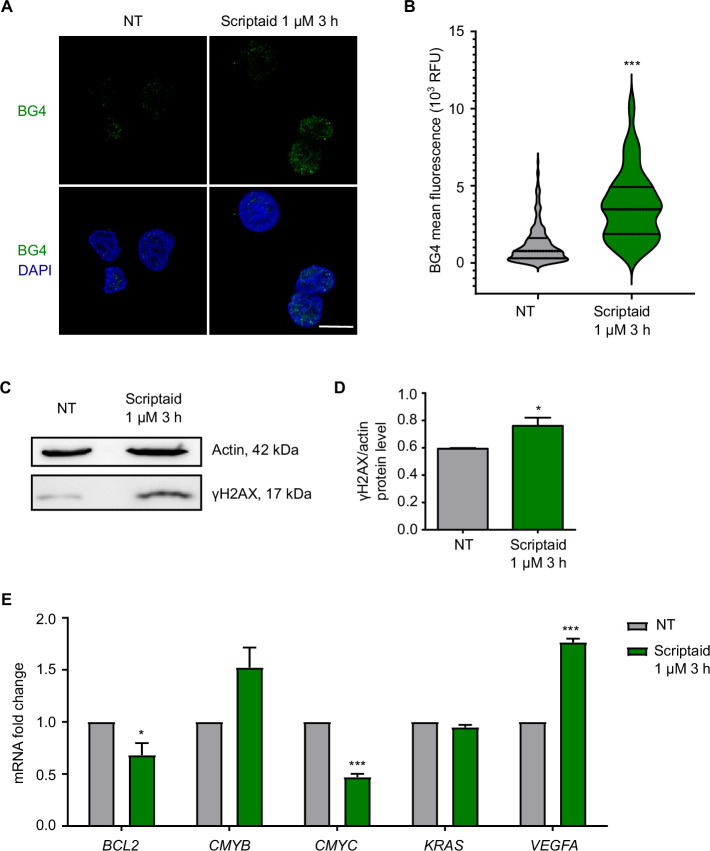
Scriptaid stabilizes G4s and induces DNA damage. (A) Immunofluorescence images of SW480 cells treated with vehicle (NT) or Scriptaid 1 µM for 3 h using the G4-selective antibody BG4. Nuclei are coloured in blue by counterstaining with DAPI. Scale bar, 10 µm. (B) Nuclear BG4 fluorescence quantification from cells in (A) by Fiji analysis (*n* > 75). (C) Western blot experiments in SW480 cells upon treatment with vehicle (NT) or Scriptaid 1 µM for 3 h to determine protein levels of a marker of DNA damage such as γH2AX and actin as housekeeping gene. Experiments were performed in biological triplicate and representative lanes are displayed. (D) Quantification of γH2AX protein levels normalized to actin (housekeeping gene) of data in (C) by ImageJ. (E) SW480 cells were treated with vehicle DMSO (NT) or Scriptaid 1 µM for 3 h and mRNA levels of several oncogenes that contain G4s in their promoters were analysed by qRT-PCR. Experiments were performed in biological triplicate.

Based on our previous study [14] that associates ribosomal G4 stabilization and inhibition of Pol I transcription, we presumed that Scriptaid could affect the stabilization properties of G4s. To investigate this hypothesis, we performed immunofluorescence experiments in SW480 cells with BG4, a G4-selective antibody. Scriptaid 1 µM for 3 h prompted a notorious increase of BG4 signal in the nucleus (figure 3A). In fact, the quantification of BG4 mean fluorescence rendered a significant increase after Scriptaid treatment, indicating that Scriptaid stabilized G4 structures (figure 3B). Such stabilization of G4s was similar to that upon treatment with the G4 ligand CX5461 1 µM for 3 h (electronic supplementary material, figure S6). As additional evidence, we analysed the DNA damage response because G4s stabilization is commonly associated with the induction of double-strand breaks in DNA [29]. To this end, we measured by Western blot a DNA damage marker such as the phosphorylation of histone H2AX on Ser-139 (γH2AX). Scriptaid 1 µM for 3 h significantly induced DNA damage (figure 3C,D; electronic supplementary material, figure S7). Importantly, these effects were confirmed in another cell line. In particular, Scriptaid 1 µM for 3 h significantly stabilized G4s and prompted the DNA damage response in melanoma A375 cells (electronic supplementary material, figure S8). Considering that Scriptaid stabilized G4s both in the nucleolus and the nucleoplasm, as well as Scriptaid induced a cellular response against double-strand breaks, we hypothesized that Scriptaid could interact with additional G4s, apart from ribosomal G4s, modulating gene expression in a broader manner. By qRT-PCR, we measured the transcriptional levels of several oncogenes including *BCL2*, *CMYB*, *CMYC*, *KRAS* and *VEGFA*, which harbour well-characterized G4s in their promoters [16]. Among these RNA polymerase II (Pol II)-transcribed genes, Scriptaid 1 µM for 3 h significantly affected the mRNA expression of *BCL2*, *CMYC* and *VEGFA* in SW480 cells (figure 3E).

### 3.5. Scriptaid interacts with ribosomal G4s *in vitro*

**Figure 4.**
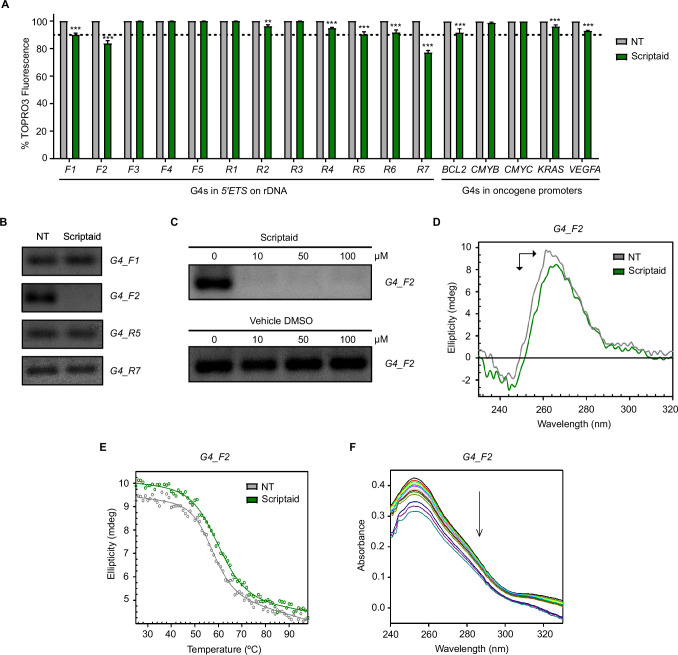
Scriptaid interacts with ribosomal G4s *in vitro*. (A) TOPRO3 FID assay with oligonucleotides including putative G4 sequences found in the *5’ETS* region of ribosomal DNA and G4s in oncogene promoters to determine the fluorescence percentage in the absence (NT) or presence of Scriptaid 10 µM. We considered interaction when the fluorescence percentage was below the threshold at 90% (dotted line). Experiments were performed in triplicate. (B) PCR stop assay to determine the effect of Scriptaid 100 µM on the stabilization of the G4-forming candidates *G4_F1*, *G4_F2*, *G4_R5*, *G4_R7*. Three independent reactions were carried out per concentration and representative lanes are shown. (C) PCR-stop assay to investigate the effect of increasing concentrations of Scriptaid or the corresponding vehicle DMSO when added to the G4-forming oligonucleotide *G4_F2*. Three independent reactions were performed per concentration and representative lanes are displayed. (D) CD spectra of the G4 formed by *G4_F2* in the absence or presence of Scriptaid 100 µM. The arrows specify the direction of movement of CD peaks after incubation with Scriptaid. (E) CD melting curve of *G4_F2* in the absence or presence of Scriptaid 100 µM. (F) UV−vis spectra of *G4_F2* upon addition of increments of Scriptaid up to 250 µM as final concentration. The arrow indicates the movement of the absorption peak upon the addition of Scriptaid.

Considering the effects described above and the similarities in chemical structure between naphthalimides and naphthalene diimides, we hypothesized that the naphthalimide Scriptaid could bind to G4s in a similar manner to naphthalene diimides [14]. To test this, we carried out TOPRO3 fluorescent intercalator displacement (FID) assays with 12 putative G4-forming sequences in the *5’ETS* region of human ribosomal DNA, as well as those in oncogene promoters including *BCL2* [30], *CMYB* [31], *CMYC* [31], *KRAS* [31] and *VEGFA* [32]. Interestingly, Scriptaid 10 µM preferentially bound to *5’ETS_F1*, *5’ETS_F2, 5’ETS_R5* and *5’ETS_R7*, decreasing the fluorescence percentage below 90% in a significant manner (figure 4A). *in vitro* DNA polymerase extension experiments with these candidate sequences only revealed a significant stalling of DNA polymerase in reactions including Scriptaid 100 µM and *5’ETS_F2* (figure 4B; electronic supplementary material, figure S9A). These data suggest that the specific binding of Scriptaid with *5’ETS_F2* G4 structure hindered the progression of DNA polymerase. Such effect was detected after incubation with Scriptaid even at 10 µM, while no inhibition was observed in reactions including DMSO as vehicle (figure 4C; electronic supplementary material, figure S9B). We confirmed that these changes were due to the binding of Scriptaid to *5’ETS_F2* G4 because the use of a mutated sequence incapable of G4 formation did not render any significant effect both in FID and DNA polymerase extension experiments (electronic supplementary material, figure S10).

CD profile of *5’ETS_F2* sequence showed a negative band at 240 nm and a positive band around 260 nm, indicating the formation of a parallel G4 (figure 4D). Upon addition of Scriptaid 100 µM, the intensity of the positive CD band decreased and the maximum was red-shifted suggesting that Scriptaid alters the conformation of *5’ETS_F2* G4 (figure 4D). However, the variations of the CD spectrum were minimal as described elsewhere [33], proving that the overall G4 folding was preserved. In fact, the absence of induced CD signal (electronic supplementary material, figure S11) indicates a mode of binding weaker than intercalation such as end-stacking or electrostatic interaction, as reported for other G4 ligands [34,35]. Moreover, CD melting experiments were carried out to assess the thermal stabilization properties of Scriptaid. The melting temperature of *5’ETS_F2* G4 was 57.2 ± 0.6°C, which increased up to 59.8 ± 0.6°C after the addition of Scriptaid 100 µM (ratio 1:10, DNA:Scriptaid), confirming the stabilization of this G4 by Scriptaid (figure 4E). In addition, *5’ETS_F2* G4 was titrated with increasing concentration of Scriptaid and followed by UV−vis spectroscopy. The complex peaked at 254 nm and displayed hypochromicity when Scriptaid was added (figure 4F). Data analysis yielded a dissociation constant (K_d_) of 622 ± 194 µM (*r*^2^ = 0.9841), thus corroborating that Scriptaid acts as a weak binder of *5’ETS_F2* G4 *in vitro*.

### 3.6. Scriptaid interacts with ribosomal G4s *in vivo*

**Figure 5.**
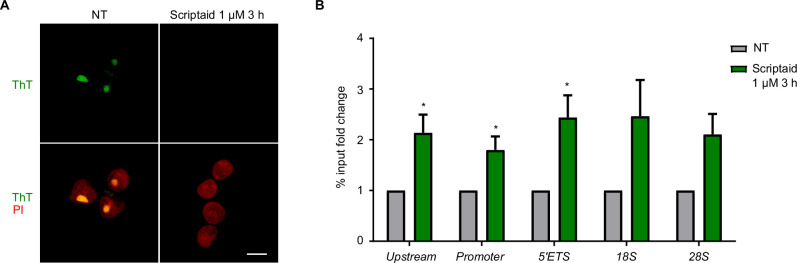
Scriptaid interacts with ribosomal G4s *in vivo*. (A) Fluorescence images of SW480 cells pre-stained with thioflavin T (ThT) and treated with vehicle (NT) or Scriptaid 1 µM for 3 h. Nuclei are in red due to counterstain with propidium iodide (PI). Scale bar, 10 µm. Experiments were performed in biological triplicate and representative images are displayed. (B) BG4 ChIP-qPCR analyses of G4 levels in SW480 cells treated with vehicle (NT) or Scriptaid 1 µM for 3 h. The different regions of ribosomal DNA that were studied are shown on the *x*-axis. Experiments were performed in biological triplicate.

The interaction of Scriptaid with *5’ETS_F2* G4 *in vitro* led us to investigate whether Scriptaid could bind to ribosomal G4s *in vivo*. For that, we performed a competition experiment *in vivo* with thioflavin T (ThT), a light-up probe of ribosomal G4s that fluoresces in the nucleolus [21]. In SW480 cells, the ThT-stained foci were lost upon treatment with Scriptaid 1 µM for 3 h, indicating that Scriptaid displaced ThT from the ribosomal G4s in a cellular environment (figure 5A). In addition, we tested whether Scriptaid 1 µM for 3 h could be altering ribosomal G4s by BG4-ChIP and qPCR using primers for different regions of the ribosomal gene body. As a consequence of Scriptaid treatment, G4 levels were significantly affected throughout the ribosomal DNA (figure 5B). It is important to note here that such effect was produced at a lower dose within the cells (1 µM) than in *in vitro* experiments (10–100 µM). Overall, these results indicate that Scriptaid interacts with G4s in a direct manner and may elicit the inhibition of rRNA synthesis by binding to G4s harboured in ribosomal DNA.

## 4. Discussion

HDACIs comprise structurally different compounds that act as anti-tumoural agents. In particular, Scriptaid is an HDACI belonging to the class of hydroxamic acid that can be synthesized in the laboratory [19]. Cytotoxic effects of Scriptaid have been reported not only on ovarian [36] and breast [37] cancer cell lines, but also on glioma [38]. Here, we have confirmed its cytotoxic potency in CRC in agreement with a previous study [39]. In fact, the IC_50_ values at micromolar range that are disclosed in the present study are in line with different works published elsewhere [12,40]. Compared to other HDACIs, Scriptaid displays cytotoxic activity in a similar manner to Vorinostat (SAHA) at micromolar concentration [41]. In contrast, trichostatin A (TSA) possesses a higher toxicity with IC_50_ values at nanomolar range, which limits its application to laboratory experiments [42].

Treatment of cancer cells with HDACIs produces pleiotropic effects, including cell cycle arrest, induction of apoptosis, and autophagy and inhibition of angiogenesis [5]. Such diverse outcomes are commonly explained by the fact that HDACs deacetylate both histone and non-histone proteins, affecting expression patterns in a broad manner. Targets of HDACIs have been extensively explored in the last decade [43], but our study is innovative in focusing on the G4-stabilization properties of Scriptaid. In the present study, we unravel that Scriptaid directly interacts with G4s on ribosomal DNA. Consequently, this action would lead to the downstream inhibition of transcription by Pol I and nucleolar stress. Part of such effects on Pol I and nucleoli may be mediated by the activity of Scriptaid as an HDACI [44–47], although the impact of acetylation changes usually requires longer. In agreement with a previous study, Scriptaid induces DNA damage [38]. Furthermore, the G4-stabilizing activity of Scriptaid is established both *in vitro* at 10−100 µM and in a cellular environment at 1 µM. This gap in concentration between *in vitro* and *in vivo* experiments could be explained considering that the experimental conditions are undoubtedly different. Within the cell, salt conditions, chromatin architecture and/or cellular state may have an impact on the results. However, to the best of our knowledge, it is the first time that an HDACI is described as a G4 ligand, paving the way for future studies.

Apart from targeting ribosomal G4s, Scriptaid probably interacts with additional G4s, which we have not deciphered yet in this work. Based on our results, Scriptaid stabilizes G4s not only in the nucleolus, but also in the nucleoplasm, and affects the transcriptional level of several oncogenes that harbour G4s in their promoters. However, our FID experiments do not reveal the interaction of Scriptaid with some of these G4s. Further investigation is required to identify whether G4s (DNA or RNA G4s) are involved in these regulation loops. Compelling research has proposed that G4s may be involved in long-distance epigenetic mechanisms, being part of an interconnected network of interactions with other biomolecules within living cells [48].

Despite growing evidence for the anti-tumoural potency of HDACIs and G4 ligands separately, only a small minority have explored its combination for cancer therapy. For instance, the G4-binding small molecule CM03 and the HDACI SAHA have been reported to display synergistic effects in pancreatic cancer [49]. Those authors propose a mechanistic model whereby SAHA inhibits HDACs, inducing chromatin relaxation and resulting in an enhanced G4 formation. Thanks to the higher levels of G4s, CM03 is able to stabilize a greater number of G4s, causing the downregulation of more G4-containing genes and a higher incidence of DSBs. However, apart from the feasible chromatin relaxation, here we suggest an active role for HDACIs in the stabilization of G4 structures. Recently, novel G4/HDAC dual-targeting compounds have been designed and synthesized by connecting the zinc-binding pharmacophore of HDACI to the G4-targeting scaffold [50]. Nevertheless, based on our results, we hypothesize that such dual-targeting of HDACs and G4s may be achievable with the HDACIs CAP group itself.

Undoubtedly, this study is the starting point for more comprehensive investigations. We have examined only one HDACI, while there is a broad group of HDACIs discovered [51] which could be explored as G4 ligands in future studies. In addition, the effects of HDACIs on different cancers are known to vary according to their anti-tumour activity, toxicity and stability [52]. Here, we have confirmed the G4 stabilization properties of Scriptaid in CRC and melanoma cells. Thus, our next steps will be focused on analysing whether the observed G4 interaction is maintained across a broader panel of different tumour types. Interestingly, the above-mentioned activity of Scriptaid is similar to other well-known G4 ligands such as naphthalene diimides [14]. Their selectivity pattern seems to be dose-dependent, but neither of the previous studies has explicitly dictated the dose–target relationship [53]. Based on the chemical similarities between the naphthalimide Scriptaid and nahphtalene diimides, it is reasonable to suppose that Scriptaid may additionally interact with other G4-containing genes. Although there is much more to be understood about the underlying mechanism of G4 targeting, further research will extend our results. Considering the anti-tumour therapeutic potential of HDACIs and G4 ligands separately, we anticipate the possibility of future therapies using the same small molecule acting both as an HDACI and as a G4 ligand.

## 5. Conclusions

We disclose that the HDACI Scriptaid stabilizes DNA G4s as an unanticipated mode of action and such activity may explain its pleiotropic effects. We reveal the interaction between Scriptaid and ribosomal G4s that interferes with RNA polymerase I transcription, leading to the induction of cytotoxicity, cell cycle arrest, nucleolar stress and DNA damage in CRC cells. Therefore, our work paves the way towards G4 targeting with HDACIs.

## Data Availability

Data sharing not applicable to this article as no datasets were generated or analysed during the current study. Supplementary material is available online [54].

## References

[1] Sharma S, Kelly TK, Jones PA. 2010 Epigenetics in cancer. Carcinogenesis **31**, 27–36. (10.1093/carcin/bgp220) 19752007 PMC2802667

[2] Li J, Hao D, Wang L, Wang H, Wang Y, Zhao Z *et al*. 2017 Epigenetic targeting drugs potentiate chemotherapeutic effects in solid tumor therapy. Sci. Rep. **7**, 4035. (10.1038/s41598-017-04406-0) 28642588 PMC5481380

[3] Ghasemi S. 2020 Cancer’s epigenetic drugs: where are they in the cancer medicines? Pharmacogenomics J. **20**, 367–379. (10.1038/s41397-019-0138-5) 31819161

[4] Frew AJ, Johnstone RW, Bolden JE. 2009 Enhancing the apoptotic and therapeutic effects of HDAC inhibitors. Cancer Lett. **280**, 125–133. (10.1016/j.canlet.2009.02.042) 19359091

[5] Li Y, Seto E. 2016 HDACs and HDAC inhibitors in cancer development and therapy. Cold Spring Harb. Perspect. Med. **6**, a026831. (10.1101/cshperspect.a026831) 27599530 PMC5046688

[6] Mann BS, Johnson JR, Cohen MH, Justice R, Pazdur R. 2007 FDA approval summary: Vorinostat for treatment of advanced primary cutaneous T-cell lymphoma. Oncologia **12**, 1247–1252. (10.1634/theoncologist.12-10-1247) 17962618

[7] Mottamal M, Zheng S, Huang TL, Wang G. 2015 Histone deacetylase inhibitors in clinical studies as templates for new anticancer agents. Molecules **20**, 3898–3941. (10.3390/molecules20033898) 25738536 PMC4372801

[8] Lee HY, Tang DW, Liu CY, Cho EC. 2022 A novel HDAC1/2 inhibitor suppresses colorectal cancer through apoptosis induction and cell cycle regulation. Chem. Biol. Interact. **352**. (10.1016/j.cbi.2021.109778) 34929181

[9] Finnin MS, Donigian JR, Cohen A, Richon VM, Rifkind RA, Marks PA, Breslow R, Pavletich NP. 1999 Structures of a histone deacetylase homologue bound to the TSA and SAHA inhibitors. Nature **401**, 188–193. (10.1038/43710) 10490031

[10] Su GH, Sohn TA, Ryu B, Kern SE. 2000 A novel histone deacetylase inhibitor identified by high-throughput transcriptional screening of a compound library. Cancer Res. **60**, 3137–3142. https://aacrjournals.org/cancerres/article/60/12/3137/506354/A-Novel-HistoneDeacetylase-Inhibitor-Identified 10866300

[11] Hutt DM *et al*. 2010 Reduced histone deacetylase 7 activity restores function to misfolded CFTR in cystic fibrosis. Nat. Chem. Biol. **6**, 25–33. (10.1038/nchembio.275) 19966789 PMC2901172

[12] Janaki Ramaiah M, Naushad SM, Lavanya A, Srinivas C, Anjana Devi T, Sampathkumar S. 2017 Scriptaid cause histone deacetylase inhibition and cell cycle arrest in HeLa cancer cells: a study on structural and functional aspects. Gene **627**, 379–386. (10.1016/j.gene.2017.06.031) 28668345

[13] Yen SF, Gabbay EJ, Wilson WD. 1982 Interaction of aromatic imides with deoxyribonucleic acid: spectrophotometric and viscometric studies. Biochemistry **21**, 2070–2076. (10.1021/bi00538a014) 7093231

[14] Sanchez-Martin V *et al*. 2021 1–12 Targeting ribosomal G-quadruplexes with naphthalene-diimides as RNA polymerase I inhibitors for colorectal cancer treatment. Cell Chem. Biol. **28**, 1590–1601.(10.1016/j.chembiol.2021.05.021) 34919843

[15] Spiegel J, Adhikari S, Balasubramanian S. 2020 The structure and function of DNA G-quadruplexes. Trends Chem. **2**, 123–136. (10.1016/j.trechm.2019.07.002) 32923997 PMC7472594

[16] Sanchez-Martin V, Lopez-Pujante C, Soriano-Rodriguez M, Garcia-Salcedo JA. 2020 An updated focus on quadruplex structures as potential therapeutic targets in cancer. Int. J. Mol. Sci. **21**, 8900. (10.3390/ijms21238900) 33255335 PMC7734589

[17] Sanchez-Martin V, Soriano M, Garcia-Salcedo JA. 2021 Quadruplex ligands in cancer therapy. Cancers **13**, 3156. (10.3390/cancers13133156) 34202648 PMC8267697

[18] Pasini A, Marchetti C, Sissi C, Cortesi M, Giordano E, Minarini A *et al*. 2017 Novel polyamine–naphthalene diimide conjugates targeting histone deacetylases and DNA for cancer phenotype reprogramming. ACS Med. Chem. Lett. **8**, 1218–1223. (10.1021/acsmedchemlett.7b00289) 29259737 PMC5733267

[19] Fleming CL, Ashton TD, Nowell C, Devlin M, Natoli A, Schreuders J *et al*. 2015 A fluorescent histone deacetylase (HDAC) inhibitor for cellular imaging. Chem. Commun. **51**, 7827–7830. (10.1039/c5cc02059j) 25853994

[20] Biffi G, Tannahill D, McCafferty J, Balasubramanian S. 2013 Quantitative visualization of DNA G-quadruplex structures in human cells. Nat. Chem. **5**, 182–186. (10.1038/nchem.1548) 23422559 PMC3622242

[21] Zhang S, Sun H, Chen H, Li Q, Guan A, Wang L *et al*. 2018 Direct visualization of nucleolar G-quadruplexes in live cells by using a fluorescent light-up probe. Biochim. Et Biophys. Acta Gen. Subj. **1862**, 1101–1106. (10.1016/j.bbagen.2018.01.022) 29410183

[22] Hänsel-Hertsch R, Spiegel J, Marsico G, Tannahill D, Balasubramanian S. 2018 Genome-wide mapping of endogenous G-quadruplex DNA structures by chromatin immunoprecipitation and high-throughput sequencing. Nat. Protoc. **13**. (10.1038/nprot.2017.150) 29470465

[23] Sanchez-Martin V *et al*. 2022 Gallic acid: a natural phenolic compound exerting antitumoral activities in colorectal cancer via interaction with G-quadruplexes. Cancers **14**, 2648. (10.3390/cancers14112648) 35681628 PMC9179882

[24] Hostettmann K. 1991 Assays for bioactivity. San Diego, CA: Academic Press.

[25] Hernandez-Verdun D. 2006 Nucleolus: from structure to dynamics. Histochem. Cell Biol. **125**, 127–137. (10.1007/s00418-005-0046-4) 16328431

[26] Pontvianne F *et al*. 2016 Identification of aucleolus-associated chromatin domains reveals a role for the nucleolus in 3D organization of the A. thaliana genome. Cell Rep. **16**, 1574–1587. (10.1016/j.celrep.2016.07.016) 27477271 PMC5279810

[27] Tajrishi MM, Tuteja R, Tuteja N. 2011 Nucleolin: the most abundant multifunctional phosphoprotein of nucleolus. Commun. Integr. Biol. **4**, 267–275. (10.4161/cib.4.3.14884) 21980556 PMC3187884

[28] Popov A, Smirnov E, Kováčik L, Raška O, Hagen G, Stixová L. 2013 Duration of the first steps of the human rRNA processing. Nucleus **4**, 134–141. (10.4161/nucl.23985) 23412654 PMC3621745

[29] Rodriguez R *et al*. 2012 Small-molecule–induced DNA damage identifies alternative DNA structures in human genes. Nat. Chem. Biol. **8**, 301–310. (10.1038/nchembio.780) 22306580 PMC3433707

[30] Agrawal P, Lin C, Mathad RI, Carver M, Yang D. 2014 The major G-quadruplex formed in the human BCL-2 proximal promoter adopts a parallel structure with a 13-nt loop in K+ solution. J. Am. Chem. Soc. **136**, 1750–1753. (10.1021/ja4118945) 24450880 PMC4732354

[31] González V, Guo K, Hurley L, Sun D. 2009 Identification and characterization of nucleolin as a c-myc G-quadruplex-binding protein. J. Biol. Chem. **284**, 23622–23635. (10.1074/jbc.m109.018028) 19581307 PMC2749137

[32] Sun D, Liu WJ, Guo K, Rusche JJ, Ebbinghaus S, Gokhale V, Hurley LH. 2008 The proximal promoter region of the human vascular endothelial growth factor gene has a G-quadruplex structure that can be targeted by G-quadruplex–interactive agents. Mol. Cancer Ther. **7**, 880–889. (10.1158/1535-7163.mct-07-2119) 18413801 PMC2367258

[33] Yadav K *et al*. 2017 Telomerase inhibition and human telomeric G-quadruplex DNA stabilization by a β-carboline–benzimidazole derivative at low concentrations. Biochemistry **56**, 4392–4404. (10.1021/acs.biochem.7b00008) 28737386

[34] Musso L, Mazzini S, Rossini A, Castagnoli L, Scaglioni L, Artali R, Di Nicola M, Zunino F, Dallavalle S. 2018 c-MYC G-quadruplex binding by the RNA polymerase I inhibitor BMH-21 and analogues revealed by a combined NMR and biochemical Approach. Biochim. Biophys. Acta Gen. Subj. **1862**, 615–629. (10.1016/j.bbagen.2017.12.002) 29229300

[35] Platella C, Mazzini S, Napolitano E, Mattio L, Beretta G, Zaffaroni N. 2021 Plant-derived stilbenoids as DNA-binding agents: from monomers to dimers. Chem Eur J **27**, 8832–8845. (10.1002/chem.202101229) 33890349 PMC8251996

[36] Takai N, Ueda T, Nishida M, Nasu K, Narahara H. 2006 A novel histone deacetylase inhibitor, Scriptaid, induces growth inhibition, cell cycle arrest and apoptosis in human endometrial cancer and ovarian cancer cells. Int. J. Mol. Med. 323–329. (10.3892/ijmm.17.2.323) 16391833

[37] Giacinti L, Giacinti C, Gabellini C, Rizzuto E, Lopez M, Giordano A. 2012 Scriptaid effects on breast cancer cell lines. J. Cell. Physiol. **227**, 3426–3433. (10.1002/jcp.24043) 22213035

[38] Sharma V, Koul N, Joseph C, Dixit D, Ghosh S, Sen E. 2010 HDAC inhibitor, scriptaid, induces glioma cell apoptosis through JNK activation and inhibits telomerase activity. J. Cell. Mol. Med. **14**, 2151–2161. (10.1111/j.1582-4934.2009.00844.x) 19583803 PMC3823006

[39] Lee EJ, Lee BB, Kim SJ, Park YD, Park J, Kim DH. 1992 Histone deacetylase inhibitor scriptaid induces cell cycle arrest and epigenetic change in colon cancer cells. Int. J. Oncol. **33**, 767–776. (10.3892/ijo_00000063) 18813790

[40] KalantarMotamedi Y, Choi RJ, Koh SB, Bramhall JL, Fan TP, Bender A. 2021 Prediction and identification of synergistic compound combinations against pancreatic cancer cells. iScience **24**, 103080. (10.1016/j.isci.2021.103080) 34585118 PMC8456050

[41] Wawruszak A, Luszczki JJ, Grabarska A, Gumbarewicz E, Dmoszynska-Graniczka M, Polberg K, Stepulak A. 2015 Assessment of interactions between cisplatin and two histone deacetylase inhibitors in MCF7, T47D and MDA-MB-231 human breast cancer cell lines: an isobolographic analysis. PLoS One **10**, e0143013. (10.1371/journal.pone.0143013) 26580554 PMC4651465

[42] You BR, Park WH. 2013 Trichostatin A induces apoptotic cell death of HeLa cells in a Bcl-2 and oxidative stress-dependent manner. Int. J. Oncol. **42**, 359–366. (10.3892/ijo.2012.1705) 23165748

[43] Buchwald M, Krämer OH, Heinzel T. 2009 HDACi – Targets beyond chromatin. Cancer Lett. **280**, 160–167. (10.1016/j.canlet.2009.02.028) 19342155

[44] Corman A, Sirozh O, Lafarga V, Fernandez-Capetillo O. 2023 Targeting the nucleolus as a therapeutic strategy in human disease. Trends Biochem. Sci. **48**, 274–287. (10.1016/j.tibs.2022.09.006) 36229381

[45] Pelletier G, Stefanovsky VY, Faubladier M, Hirschler-Laszkiewicz I, Savard J, Rothblum LI, Côté J, Moss T. 2000 Competitive Recruitment of CBP and Rb-HDAC Regulates UBF Acetylation and Ribosomal Transcription. Mol. Cell **6**, 1059–1066. (10.1016/s1097-2765(00)00104-0) 11106745

[46] Houston R *et al*. 2020 Acetylation-mediated remodeling of the nucleolus regulates cellular acetyl-CoA responses. PLoS Biol. **18**, 1–34. (10.1371/journal.pbio.3000981) PMC7728262 33253182

[47] Murayama A *et al*. 2008 Epigenetic control of rDNA loci in response to intracellular energy status. Cell **133**, 627–639. (10.1016/j.cell.2008.03.030) 18485871

[48] Robinson J, Raguseo F, Nuccio SP, Liano D, Di Antonio M. 2021 DNA G-quadruplex structures: more than simple roadblocks to transcription? Nucleic Acids Res. **49**, 8419–8431. (10.1093/nar/gkab609) 34255847 PMC8421137

[49] Ahmed AA, Neidle S. 2020 A G-quadruplex-binding small molecule and the HDAC inhibitor SAHA (vorinostat) act synergistically in gemcitabine-sensitive and resistant pancreatic cancer cells. Molecules **25**, 5407. (10.3390/molecules25225407) 33227941 PMC7699281

[50] Jiang XC *et al*. 2022 Discovery of a novel G-quadruplex and histone deacetylase (HDAC) dual-targeting agent for the treatment of triple-negative breast cancer. J. Med. Chem. **65**, 12346–12366. (10.1021/acs.jmedchem.2c01058) 36053318

[51] Eckschlager T, Plch J, Stiborova M, Hrabeta J. 2017 Histone deacetylase inhibitors as anticancer drugs. Int. J. Mol. Sci. **18**, 1414. (10.3390/ijms18071414) 28671573 PMC5535906

[52] Xu WS, Parmigiani RB, Marks PA. 2007 Histone deacetylase inhibitors: molecular mechanisms of action. Oncogene **26**, 5541–5552. (10.1038/sj.onc.1210620) 17694093

[53] Pirota V, Nadai M, Doria F, Richter SN. 2019 Naphthalene diimides as multimodal G-quadruplex-selective ligands. Molecules **24**. (10.3390/molecules24030426) PMC6384834 30682828

[54] Sanchez-Martin V, Ruzic D, Tello-Lopez MJ, Ortiz-Morales A, Murciano-Calles J, Soriano M, Nikolic K, Garcia-Salcedo JA. 2025 Supplementary material from: The histone deacetylase inhibitor Scriptaid targets G-quadruplexes. Figshare. (10.6084/m9.figshare.c.7644194)39965659

